# Th1 cells are dispensable for primary clearance of *Chlamydia* from the female reproductive tract of mice

**DOI:** 10.1371/journal.ppat.1010333

**Published:** 2022-02-23

**Authors:** Jordan A. Rixon, Claire E. Depew, Stephen J. McSorley

**Affiliations:** Center for Immunology and Infectious Diseases, Department of Anatomy, Physiology and Cell Biology, School of Veterinary Medicine, University of California Davis, Davis, California, United States of America; Duke University School of Medicine, UNITED STATES

## Abstract

Protective immune responses to *Chlamydia* infection within the female reproductive tract (FRT) are incompletely understood. MHC class II-restricted CD4 Th1 responses are believed to be vital for bacterial clearance due to their capacity to secrete IFN-γ, but an essential requirement for T-bet-expressing Th1 cells has yet to be demonstrated in the mouse model of *Chlamydia* infection. Here, we investigated the role of T-bet and IFN-γ in primary clearance of *Chlamydia* after FRT infection. Surprisingly, IFN-γ producing CD4 T cells from the FRT expressed low levels of T-bet throughout infection, suggesting that classical T-bet-expressing Th1 cells are inefficiently generated and therefore unlikely to participate in bacteria clearance. Furthermore, mice deficient in T-bet expression or with a CD4-specific T-bet deficiency cleared FRT infection similarly to wild-type controls. T-bet-deficient mice displayed significant skewing of FRT CD4 T cells towards Th17 responses, demonstrating that compensatory effector pathways are generated in the absence of Th1 cells. In marked contrast, IFN-γ-, and IFN-γR-deficient mice were able to reduce FRT bacterial burdens, but suffered systemic bacterial dissemination and 100% mortality. Together, these data demonstrate that IFN-γ signaling is essential to protect mice from fatal systemic disease, but that classical T-bet-expressing Th1 cells are non-essential for primary clearance within the FRT. Exploring the protective contribution of Th1 cells versus other CD4 effector lineages could provide important information for the generation of new *Chlamydia* vaccines.

## Author summary

The production of IFN-γ by CD4 Th1 cells is thought to be critical for the clearance of *Chlamydia* from the female reproductive tract (FRT), but this has not been formally tested. Here we demonstrate that T-bet+ Th1 cells are not essential for effective *Chlamydia* clearance. Furthermore, the impact of IFN-γ deficiency or depletion is largely observed as a failure to control bacterial dissemination, rather than clearance from the FRT. Together, these data suggest that different immunological mechanisms are responsible for restraining systemic spread of bacteria versus FRT control. Defining alternative non-Th1 CD4 effector mechanisms that are responsible for controlling *Chlamydia* replication within the FRT could be foundational for future vaccine development.

## Introduction

*Chlamydia trachomatis* is a gram-negative bacterium with an atypical obligate intracellular developmental cycle wherein replication occurs primarily in epithelial cells of the urogenital tract [1,2]. This chronic bacterial infection can initiate marked pathology at this mucosal surface and eventually lead to severe reproductive problems in otherwise healthy individuals [3]. In 2018, over 1.7M cases of *Chlamydia* infection were reported to the Centers for Disease Control and Prevention, with almost two-thirds of these cases found in young adults (ages 15–24 years) [4]. *Chlamydia* infections are often asymptomatic and are therefore difficult to detect and treat as part of a public health approach to reducing disease incidence [4]. Untreated *Chlamydia* infection can often lead to pelvic inflammatory disease, ectopic pregnancy, and sterility [5,6]. Given the high incidence and impact of this disease on the US population, development of a *Chlamydia* vaccine is now a public health priority [7].

Initial attempts at developing human *Chlamydia* vaccines were derailed by the detection of increased pathology in some vaccinated individuals, impeding additional human trials of vaccine candidates [8]. Despite the fact that these problems occurred almost 60 years ago, an effective *Chlamydia* vaccine remains elusive today [7,8]. Although much has been learned about *Chlamydia* pathogenesis and host immunity during this time [5,9–11], deeper understanding of the mechanisms of immune clearance from the reproductive tract are needed to assist vaccine development. Central to these efforts is the use of mouse models of *Chlamydia* infection which recapitulate many of the central features of human disease [10]. In one common model, inbred mice are infected with *C*. *muridarum*, a mouse pathogen that is delivered vaginally and naturally ascends the reproductive tract to cause significant pathology [10]. This experimental approach in mice recapitulates key features of human disease and has been widely used to study the immune response to *Chlamydia* infection [3,10].

Foundational work from several laboratories has determined that CD4 T cells are the critical lymphocyte population that orchestrate clearance of *Chlamydia* infection from the female reproductive tract [12–14]. Severe Combined Immune Deficient (SCID)-, RAG-deficient-, TCR-α-, and MHC class-II-deficient mice all fail to resolve a primary vaginal infection with *C*. *muridarum* [13,15,16]. Thus, functioning MHC class-II restricted CD4 T cell responses are the primary requirement for bacterial clearance. In marked contrast, gene-deficient mice lacking MHC class-I or B cells resolve primary *Chlamydia* infection [13,17], although systemic infection has been detected in B cell-deficient mice before clearance occurs [18,19].

The central role of CD4 T cells in combating any microbial infection relies on the capacity to develop “helper” activity, where they assist macrophages, neutrophils, B cells, CD8 T cells, or mast cells/eosinophils to kill particular classes of pathogen [20]. After infection, naïve CD4 T cells mature into Th1, Th2, Th17, Tfh, or Treg lineages that can help or impede other immune cells to kill microbes. In the case of *Chlamydia* infection, it is widely believed that CD4 Th1 cells are the major effector lineage that orchestrates bacterial clearance from the reproductive tract [10,11,21–23]. Th1 cells are often required to coordinate the immune response against intracellular pathogens via their capacity to activate macrophages through secretion of IFN-γ [24]. Th1 cells are defined by their expression of the T-box transcription factor TBX21 (T-bet), which controls expression of IFN-γ and related genes [25]. In the *C*. *muridarum* mouse model, IFN-γ has been demonstrated to play a vital role in host control of infection [26,27]. Additionally, several human studies have correlated the capacity to generate an IFN-γ responses during *Chlamydia trachomatis* infection with a lower risk of reinfection [28,29]. Together, these animal and human data provide a simple model for understanding *Chlamydia* clearance in human and murine infection, wherein Th1 cell production of IFN-γ allows the control of bacterial growth within the FRT. Despite the attraction of this Th1 cell-centric view, there are some obvious caveats to the model as it pertains to *Chlamydia* immunity.

First, published studies have reported uncontrolled growth of certain intracellular pathogens in T-bet-deficient mice in the absence of Th1 cells [30–32], but this has not yet been studied in the *Chlamydia* model. Second, looking closely at published data, mice lacking IFN-γ do not always exhibit the same level of severe deficiency observed in MHC-class-II-deficient mice [26,27,33]. Indeed, *Chlamydia* shedding from the FRT is often reduced by multiple logs from peak infection in these mice, suggesting that IFN-γ-independent mechanisms play a major role in bacterial clearance. Third, a number of recent reports have uncovered a potential role for non-Th1 cells in *Chlamydia* immunity, or in tissue repair after infection, including Type-II immune cells [34,35], Th17 cells [36,37], and CD4 T cells secreting IL-13 [38]. Furthermore, IFN-γ can also be secreted by some of these non-Th1 (T-bet-negative) CD4 T cell populations [39]. It is therefore vitally important to clearly define the contribution of CD4 T-bet^+^ Th1 cells to *Chlamydia* clearance in the mouse model.

In this study, we examined the role of IFN-γ versus T-bet in reproductive tract immunity against *Chlamydia muridarum*. Contrary to expectations, T-bet expression was low and transient in all CD4 T cells within the reproductive tract and draining iliac lymph nodes of *Chlamydia*-infected mice, despite the fact that high levels of IFN-γ production were detected. Furthermore, loss of T-bet expression in CD4 T cells, or in other host cells, did not completely eliminate IFN-γ production or impede the ability of mice to clear *Chlamydia* infection from the FRT. Interestingly, CD4 T cells in T-bet-deficient mice displayed a profound shift towards Th17 responses, suggesting compensation between these two CD4 effector subsets in the control of *Chlamydia* infection. Despite the lack of an essential requirement for Th1 cells, mouse models deficient in IFN-γ or IFN-γR displayed fatal systemic infection. Additionally, T-bet-deficient mice administered depleting anti-IFN-γ also experienced bacterial dissemination and mortality, indicating that IFN-γ is essential for resolving systemic infection, regardless of which effector mechanisms are operational in the FRT. Together, these data highlight the importance of non-Th1 cell secretion of IFN-γ outside the FRT to control systemic disease and suggest the potential for compensatory CD4 effector responses for bacterial clearance within the FRT.

## Results

### Chlamydia infection of the FRT induces IFN-γ-producing CD4 T cells

**Fig 1:**
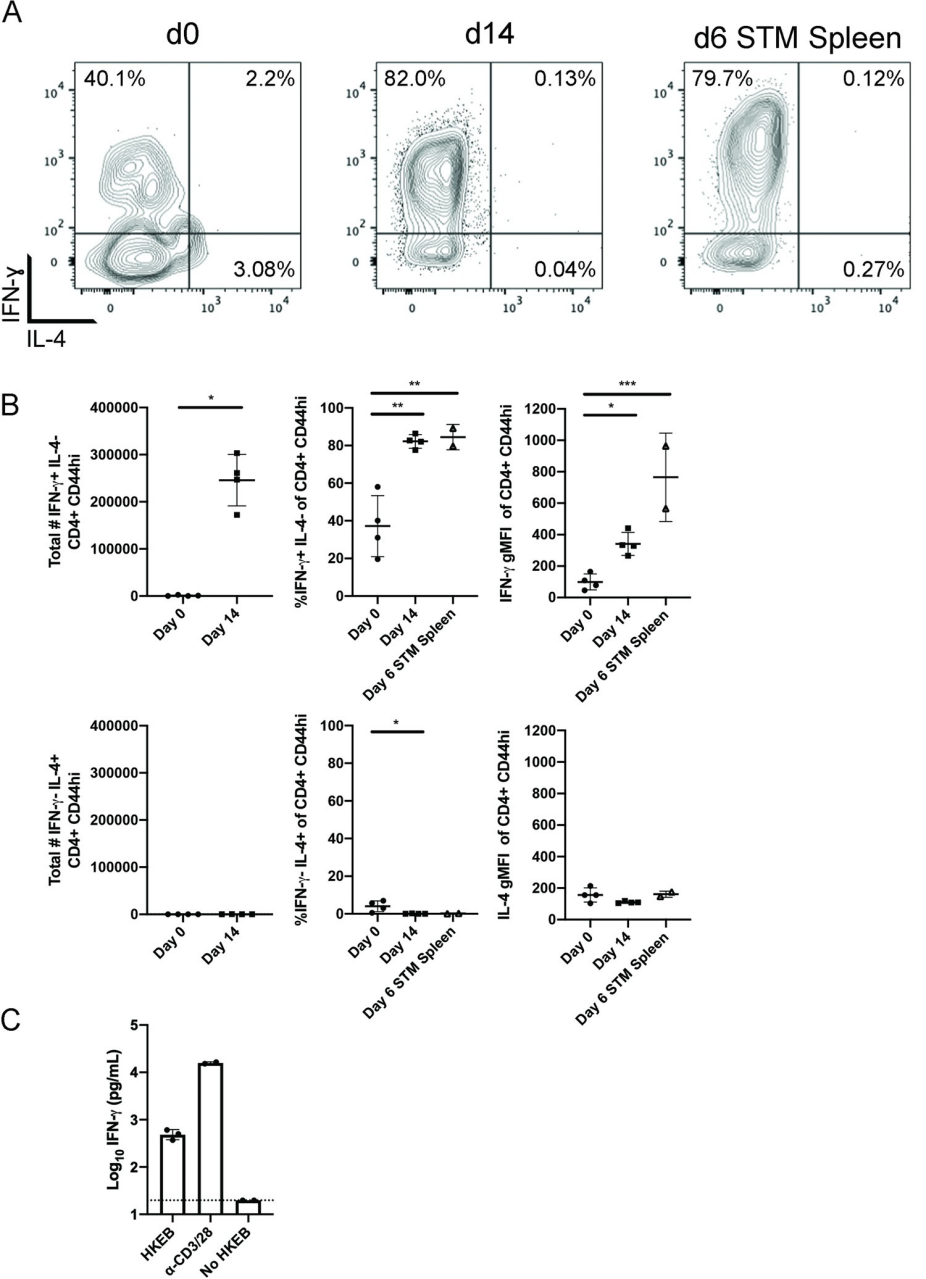
CD4 T cells respond during primary FRT infection in wild-type mice and have a strong IFN- ɣ signature. Mice either received 2.5mg Depo-Provera 7 days before infection with 1x10^5^ IFU *Chlamydia muridarum* i.vag. or 5x10^5^ CFU *Salmonella* i.v. Lymphocytes were isolated from the FRT on day 14 post infection for *Chlamydia*-infected mice or from the spleen day 6 post *Salmonella* infection and stimulated with PMA/ionomycin in the presence of Brefeldin A before staining for flow cytometry. n = 4 for naïve and *Chlamydia* groups, n = 2 for *Salmonella* group. (A) Example flow cytometry plots of IFN-ɣ and IL-4 expression gated on CD4+ CD44^hi^ cells. (B) Summary of flow analysis. (C) IFN-ɣ ELISA results for CD4 T cells isolated from the FRT of *Chalmydia* infected mice 15 days post infection and stimulated with HKEBs (Heat-Killed Elementary Bodies), controls stimulated with α-CD3 and α-CD28, or controls that did not receive HKEBs. n = 3 for HKEB stimulation and n = 2 for control groups due to low cell recovery from the third FRT. Results (B and C) are shown as mean +/- SD.

Previous work has established that IFN-γ is a common component of the CD4 T cell response to *Chlamydia* infection of the FRT [10]. We initially sought to confirm this finding by examining the production of IFN-γ by CD4 T cells in C57BL/6 mice infected vaginally with *C*. *muridarum*. CD4 T cells recovered from the FRT at day 14 post vaginal infection were assessed by flow cytometry following brief PMA/ionomycin stimulation in the presence of Brefeldin A. As expected, CD4 T cells in the FRT of *Chlamydia*-infected mice produced significant amounts of IFN-γ, but negligible IL-4 (Fig 1A and 1B). Indeed, CD4 T cell production of IFN-γ to both *Chlamydia* and *Salmonella* infection was similar (Fig 1A and 1B), reinforcing the concept that both of these bacterial infections drive strong Th1 responses. This was reflected in increased percentages and total numbers of IFN-γ+ IL-4- activated CD4 T cells, as well as increased staining intensity for IFN-γ, while staining for IL-4 was minimal. Additionally, at 15 days post infection, CD4 T cells isolated from the FRT of *Chlamydia*-infected mice produced significant levels of IFN-γ after ex vivo stimulation with heat-killed *C*. *muridarum* elementary bodies (HKEBs) (Fig 1C). Thus, vaginal *Chlamydia* infection drives robust CD4 IFN-γ production within the FRT.

### Most IFN-ɣ-producing CD4 T cells responding to Chlamydia lack T-bet expression

**Fig 2:**
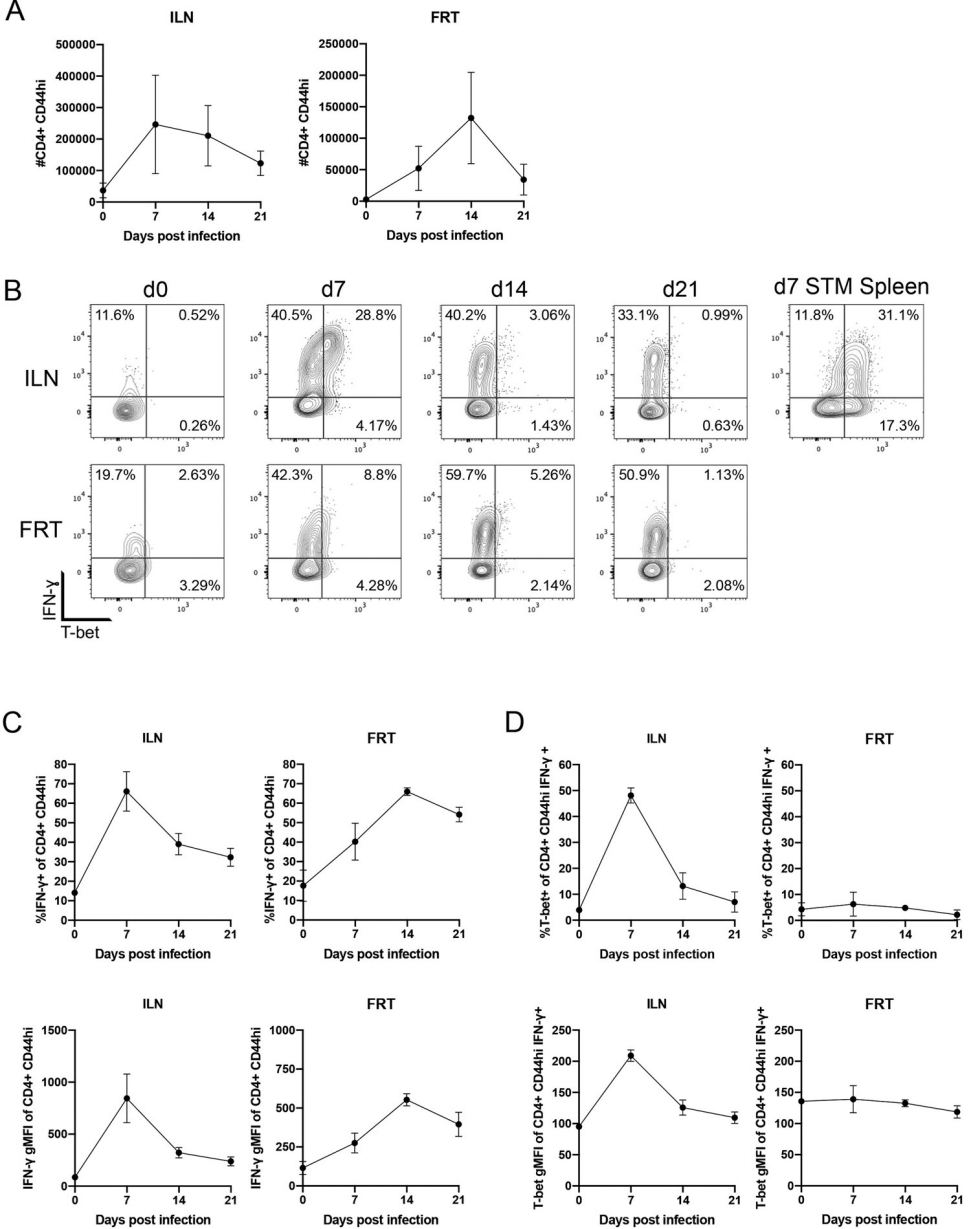
IFN-ɣ Producing Th1 cells in the FRT and draining lymph node during primary infection do not express high levels of T-bet. Mice received 2.5mg Depo-Provera 7 days before infection with 1x10^5^ IFU *Chlamydia muridarum* i.vag. or 5x10^5^ CFU *Salmonella* i.v. Lymphocytes were isolated from the FRT or ILN for *Chlamydia*-infected mice the spleen for *Salmonella* infection and stimulated with PMA/ionomycin in the presence of Brefeldin A before staining for flow cytometry. n = 3 for each group. Infections were staggered so all time points were processed on the same day. (A) Total number of CD4+CD44^hi^ cells in the ILN and FRT. (B) IFN-ɣ and T-bet expression in CD4+CD44^hi^ cells. (C) IFN-ɣ production of activated CD4s and geometric MFI of IFN-ɣ expression. (D) T-bet expression in IFN-ɣ+CD4+CD44hi cells and geometric MFI. Data is representative of two experiments. Results are shown as mean ± SD.

Since Th1 cells are defined by the expression of the master transcription factor T-bet (*Tbx21*) [25], we hypothesized that IFN-γ-producing CD4 T cells responding to *Chlamydia* infection would express T-bet. Activated CD44^+^ CD4 T cells initially expanded in the ILN and could be detected infiltrating the FRT during the first week of infection (Fig 2A). As the bacteria were cleared from the FRT during the second and third week of infection, the number of these responding CD4 T cell declined (Fig 2A). In the ILN, CD4 T cell production of IFN-γ peaked at day 7 post-infection, while the peak of IFN-γ production in the FRT occurred later, at day 14 (Fig 2B and 2C). Again, the magnitude of this CD4 IFN-γ response approximated the response of CD4 Th1 cells in *Salmonella*-infected mice (Fig 2B). However, IFN-γ-producing CD4 T cells in *Chlamydia*-infected mice expressed low levels of T-bet at all time points in the ILN, with only a transient signal detected at day 7 during the peak of the IFN-γ response (Fig 2B and 2D). More strikingly, in the FRT, IFN-γ-producing CD4+ T cells exhibited little to no T-bet expression over the full course of primary clearance of bacteria from the tissue (Fig 2B and 2D). In marked contrast, robust T-bet expression was detected in IFN-γ-producing CD4 T cells from *Salmonella*-infected mice (Fig 2B). Together, these data suggest that CD4 T cell IFN-γ production during *Chlamydia* infection is largely T-bet-independent and contrasts sharply with the response to *Salmonella* infection.

### Lack of T-bet expression does not impair resolution of primary Chlamydia infection

**Fig 3:**
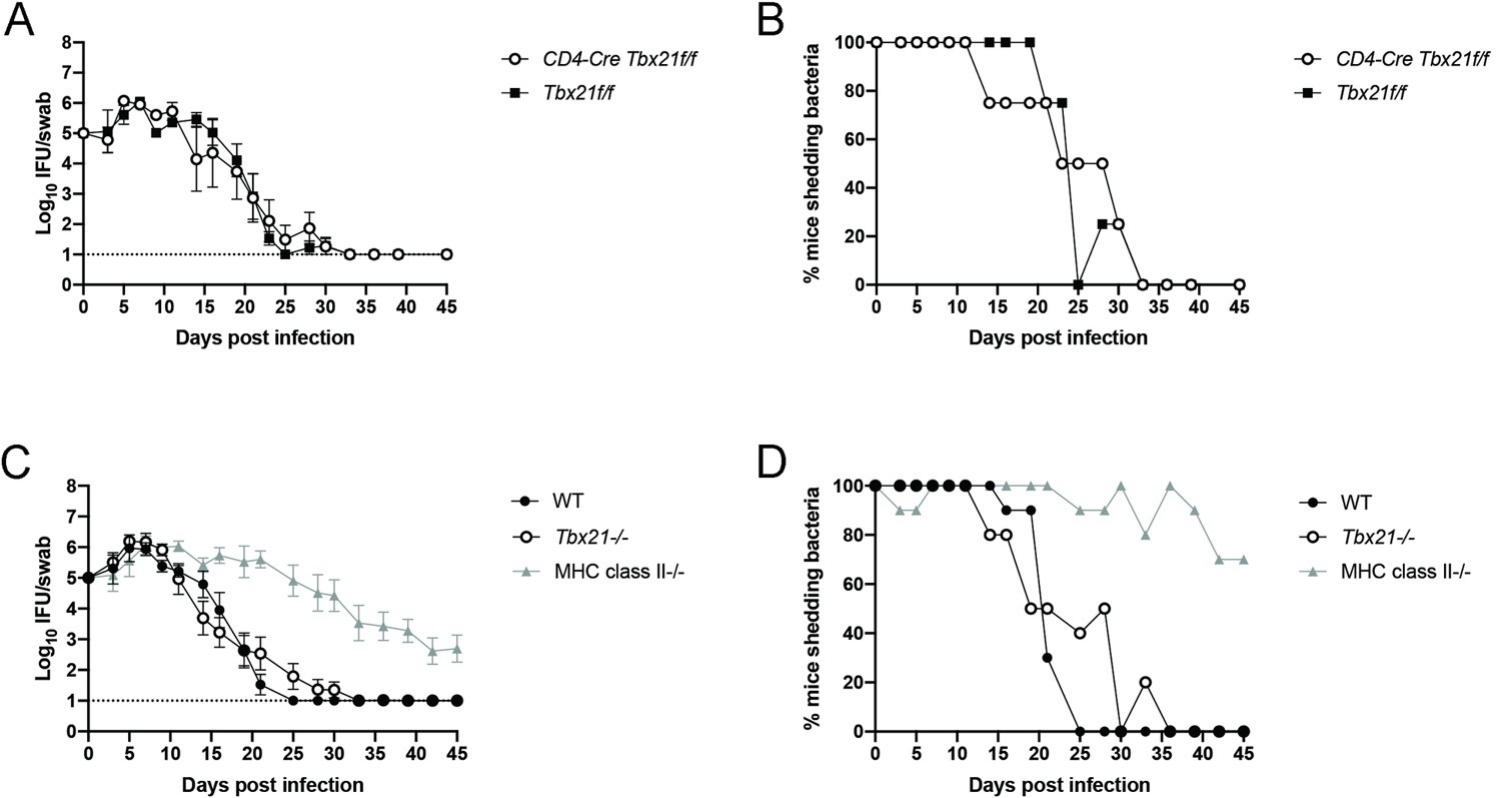
Expression of the Th1 master transcription factor T-bet is not required for clearance from the reproductive tract. Mice received 2.5mg Depo-Provera 7 days before infection with 1x10^5^ IFU *Chlamydia muridarum* i.vag. (A) IFU per vaginal swab over time. n = 4 for both groups. (B) Percent of mice from A with culture-positive swabs. (C) IFU per vaginal swab over time. Results are from two pooled experiments with a total of 10 mice per group. (D) Percent of mice from C with culture-positive swabs. (A) and (C) are shown as mean ± SEM.

Given the virtual absence of T-bet expression among *Chlamydia*-specific CD4 T cells within the FRT of infected mice, we explored whether T-bet expression was required to resolve *Chlamydia* infection. We initially utilized a Cre-loxP system to selectively delete T-bet in CD4 T cells by crossing CD4-Cre mice to a *Tbx21* floxed mouse line. Consistent with the absence of CD4 T-bet expression in the FRT (Fig 2), CD4-Cre *Tbx21*f/f mice resolved *Chlamydia* infection with similar kinetics to Cre-negative littermate controls (Fig 3A). The proportion of mice shedding bacteria at any given point during *Chlamydia* infection was also comparable between CD4-Cre *Tbx21*f/f mice and littermate controls (Fig 3B). These data conclusively demonstrate that Th1 cells are not required to resolve primary infection with *C*. *muridarum*. Since T-bet is also expressed by other immune cell lineages, such as ILC1 and NK cells [39], we explored whether T-bet expression in any cell population is necessary for resolving *Chlamydia* infection. Again, mice deficient for T-bet expression in all cell types resolved FRT *Chlamydia* infection, while mice lacking MHC class-II-restricted T cells were severely deficient in clearing bacteria (Fig 3C). This efficient FRT clearance was evident whether monitoring bacterial shedding or the proportion of mice shedding bacteria at any given point (Fig 3D). Consistent with previous reports [40,41], these same T-bet deficient mouse lines displayed a marked deficiency in resolving *Salmonella* infection and had an obvious reduction in IFN-γ production (S1 Fig). These data uncover an important difference between *Chlamydia* and *Salmonella* infection models. T-bet and Th1 cells are required to resolve primary *Salmonella* infection while T-bet expression is not necessary for primary clearance of *C*. *muridarum* infection from the FRT.

### Chlamydia-infected T-bet deficient mice retain IFN-ɣ-producing CD4 T cells but display enhanced Th17 responses

**Fig 4:**
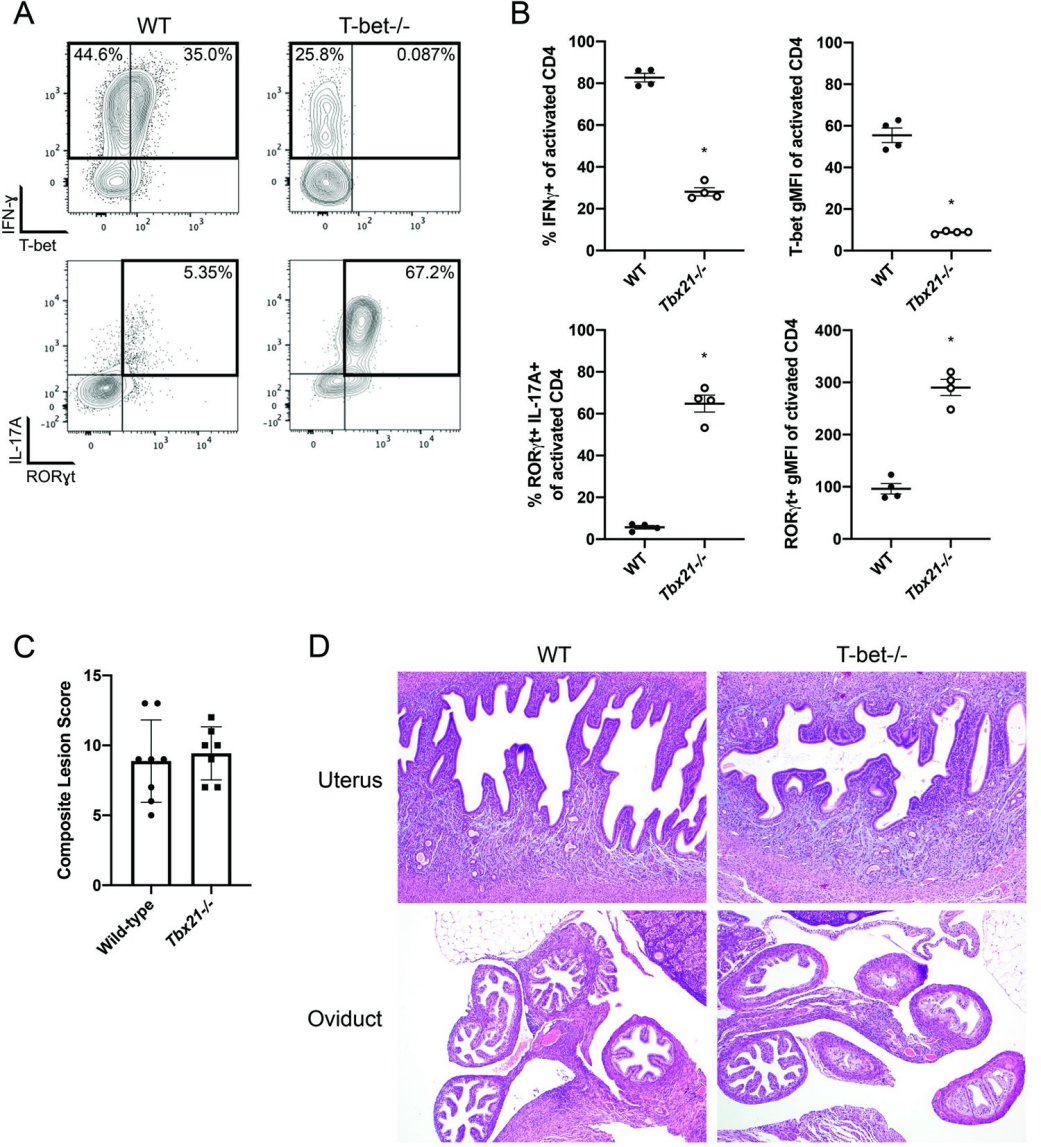
T-bet deficient mice make T-bet independent IFN-ɣ and shift towards Th17 responses. Mice received 2.5mg Depo-Provera 7 days before infection with 1x10^5^ IFU *Chlamydia muridarum* i.vag. (A) 17 days post infection lymphocytes were isolated and stimulated with PMA/ionomycin and Brefeldin A and stained for flow cytometry. n = 4 for both groups. Plots are gated on CD44^hi^ CD62L^lo^ CD4 T cells. Results are representative of two experiments. (B) Summary plots from (A). (C) Summary of composite lesion scores for wild-type and *Tbx21-/-* FRT tissue sections on day 61 post infection. Graph comprises two experiments, total n = 8 for both groups though one mouse from *Tbx21-/-* group was excluded due to suspected imperforate vagina and non-productive infection. p = 0.549, Mann-Whitney test. (D) Example histology slides from part (C).

Given the capacity of T-bet-deficient mice to efficiently resolve C*hlamydia* infection, we examined the effector response of CD4 T cells in these mice in more detail. Although CD4 T cells from T-bet-deficient mice displayed reduced capacity for IFN-γ production compared to wild-type mice, IFN-γ production was retained in 20–40% of CD4 T cells (Fig 4A and 4B). A similar reduction in IFN-γ producing capacity was observed in CD8 T cells (S2 Fig). While wild-type mice had barely detectable Th17 responses (Fig 4A and 4B), CD4 T cells in *Chlamydia*-infected T-bet-deficient mice displayed high levels of RORγt and IL-17A, comprising 60–70% of all CD4 T cells (Fig 4A and 4B). This marked shift towards Th17 development in T-bet deficient mice was confirmed by bulk RNA-sequencing comparing sorted CD4 T cells from the FRT of wild-type and T-bet-deficient mice. T-bet-deficient samples exhibited significant downregulation of RNA for Th1 markers compared to wild-type mice (Tables 1 and S1). Additionally, notable upregulation of Th17 markers *Il17a* and *Il23r* was detected, although RORγt (gene name *Rorc*) was not significantly altered (Tables 1 and S1). This compensatory Th17 response is reminiscent of IFN-γ-deficient mice where a previous report documented increased Th17 activity and corresponding tissue damage [37]. To determine if the Th17 shift in T-bet deficient mice was a pathological response, we analyzed the FRTs of mice at day 61 post-infection for signs of acute or chronic inflammation, fibrosis, erosion or ulceration, and luminal dilation. Surprisingly, the composite lesion scores and histology of T-bet deficient mice did not significantly differ from wild-type mice (Fig 4C and 4D). In summary, CD4 T cells in *Chlamydia*-infected T-bet deficient mice show a perturbed immune response with marked inhibition of IFN-γ responses and a prominent shift toward a dominant Th17 response, however this did not increase FRT pathology during primary infection.

**Table 1:** Selected gene expression analysis of *Tbx21*-deficient CD4+ T cells compared to wild-type. CD4+ T cells were FACS sorted from FRTs 17 days post infection and processed for bulk RNA sequencing. Table shows differential gene expression analysis of selected markers for Th1, Th2 and Th17 responses in *Tbx21-/-* samples compared to wild-type. p<0.05 are shown in bold. Average expression is across all samples in log2 counts per million reads. Adjusted p-value is adjusted with the Benjamini-Hochberg false discovery rate.

Gene name	Log2 Fold Change	Average Expression	Adjusted p Value	Gene description
**Th1-associated**				
*Tbx21*	-3.79	5.67	**2.79E-07**	T-box 21 [Source:MGI Symbol;Acc:MGI:1888984]
*Stat1*	1.04	8.31	**0.0006**	signal transducer and activator of transcription 1 [Source:MGI Symbol;Acc:MGI:103063]
*Stat4*	-0.42	7.06	0.2952	signal transducer and activator of transcription 4 [Source:MGI Symbol;Acc:MGI:103062]
*Ifng*	-1.91	8.32	**4.28E-07**	interferon gamma [Source:MGI Symbol;Acc:MGI:107656]
*Ifngr2*	2.96	2.81	**0.0036**	interferon gamma receptor 2 [Source:MGI Symbol;Acc:MGI:107654]
*Il12rb2*	-4.89	6.72	**1.12E-10**	interleukin 12 receptor, beta 2 [Source:MGI Symbol;Acc:MGI:1270861]
*Ccr5*	-3.63	6.14	**1.07E-06**	chemokine (C-C motif) receptor 5 [Source:MGI Symbol;Acc:MGI:107182]
*Cxcr3*	-6.72	5.09	**5.07E-08**	chemokine (C-X-C motif) receptor 3 [Source:MGI Symbol;Acc:MGI:1277207]
**Th17-associated**				
*Rorc*	3.1	0.59	0.1547	RAR-related orphan receptor gamma [Source:MGI Symbol;Acc:MGI:104856]
*Stat3*	0.05	9.53	0.9387	signal transducer and activator of transcription 3 [Source:MGI Symbol;Acc:MGI:103038]
*Il17a*	5.48	5.43	**0.0102**	interleukin 17A [Source:MGI Symbol;Acc:MGI:107364]
*Il22*	0.59	2.58	0.8698	interleukin 22 [Source:MGI Symbol;Acc:MGI:1355307]
*Il23r*	2.54	3.13	**0.0488**	interleukin 23 receptor [Source:MGI Symbol;Acc:MGI:2181693]
**Th2-associated**				
*Gata3*	0.41	6.99	0.4171	GATA binding protein 3 [Source:MGI Symbol;Acc:MGI:95663]
*Il5*	3.97	-0.7	0.1088	interleukin 5 [Source:MGI Symbol;Acc:MGI:96557]
*Il13*	2.91	0.03	0.2837	interleukin 13 [Source:MGI Symbol;Acc:MGI:96541]

### IFN-γ signaling is required to prevent fatal dissemination of Chlamydia

**Fig 5:**
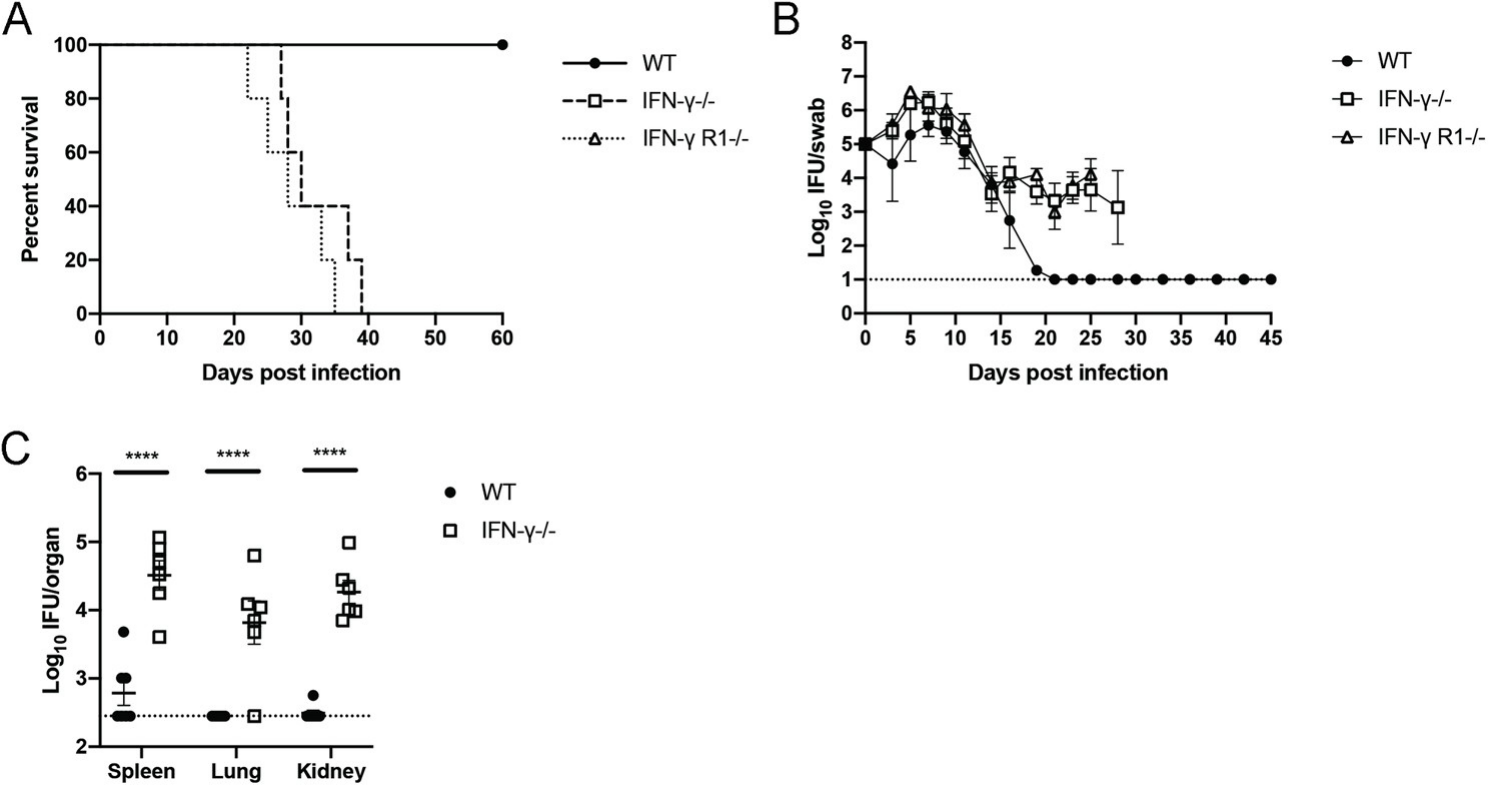
IFN-γ-deficient mice die from systemic disease, but clear the majority of FRT infection. Mice received 2.5mg Depo-Provera 7 days before infection with 1x10^5^ IFU *Chlamydia muridarum* i.vag. (A) Survival curve. (B) IFU per vaginal swab over time of mice from (A). IFN-ɣ-/- and IFN-ɣ Receptor-/- data was truncated at days 28 and 25 respectively when the number of mice surviving dropped below 50%. n = 5 for all groups in (A) and (B). Results are shown as mean with SEM. (C) Bacterial load per organ on day 14 (2-way ANOVA). Data is compiled from two separate experiments with n = 3 and 4 per group, though one IFN-ɣ-/- mouse died prior to day 14 and could not be included. **** = p<0.0001.

Our data show that non-Th1 CD4 T cells secrete IFN-γ in the FRT during *Chlamydia* infection (Fig 2B and 2D). In order to understand the contribution of these CD4 T cells and other non-CD4 sources of IFN-γ during *Chlamydia* infection, we infected IFN-γ- and IFN-γR1-deficient mice vaginally with *C*. *muridarum*. In accordance with a key role for IFN-γ signaling in *Chlamydia* defense, the mortality rate for both gene-deficient mouse lines was 100%, with median survival of 30 days for IFN-ɣ-, and 28 days for IFN-ɣR1-deficient mice (Fig 5A). Interestingly, FRT shedding was similar between wild-type, IFN-γ-, and IFN-γR1-deficient mice during the first 2 weeks of infection (Fig 5B). Indeed, initial FRT infection was reduced by at least 2 orders of magnitude in the complete absence of IFN-γ signaling (Fig 5B). However, after this initial period, FRT shedding plateaued in the absence of IFN-γ signaling, coincident with bacterial dissemination to systemic tissues (Fig 5C). Thus, despite the ability of mice to control *Chlamydia* infection for 2 weeks in in the FRT without Th1 cells or IFN-γ, IFN-γ signaling remains an essential component of *Chlamydia* immunity in this mouse model. However, the pattern of infection observed in these studies would suggest that the predominant role of IFN-γ is to prevent bacterial dissemination and death, rather than resolve local infection within the FRT.

### IFN-γ production contributes to resolution of systemic Chlamydia infection in the absence of Th1 cells

**Fig 6:**
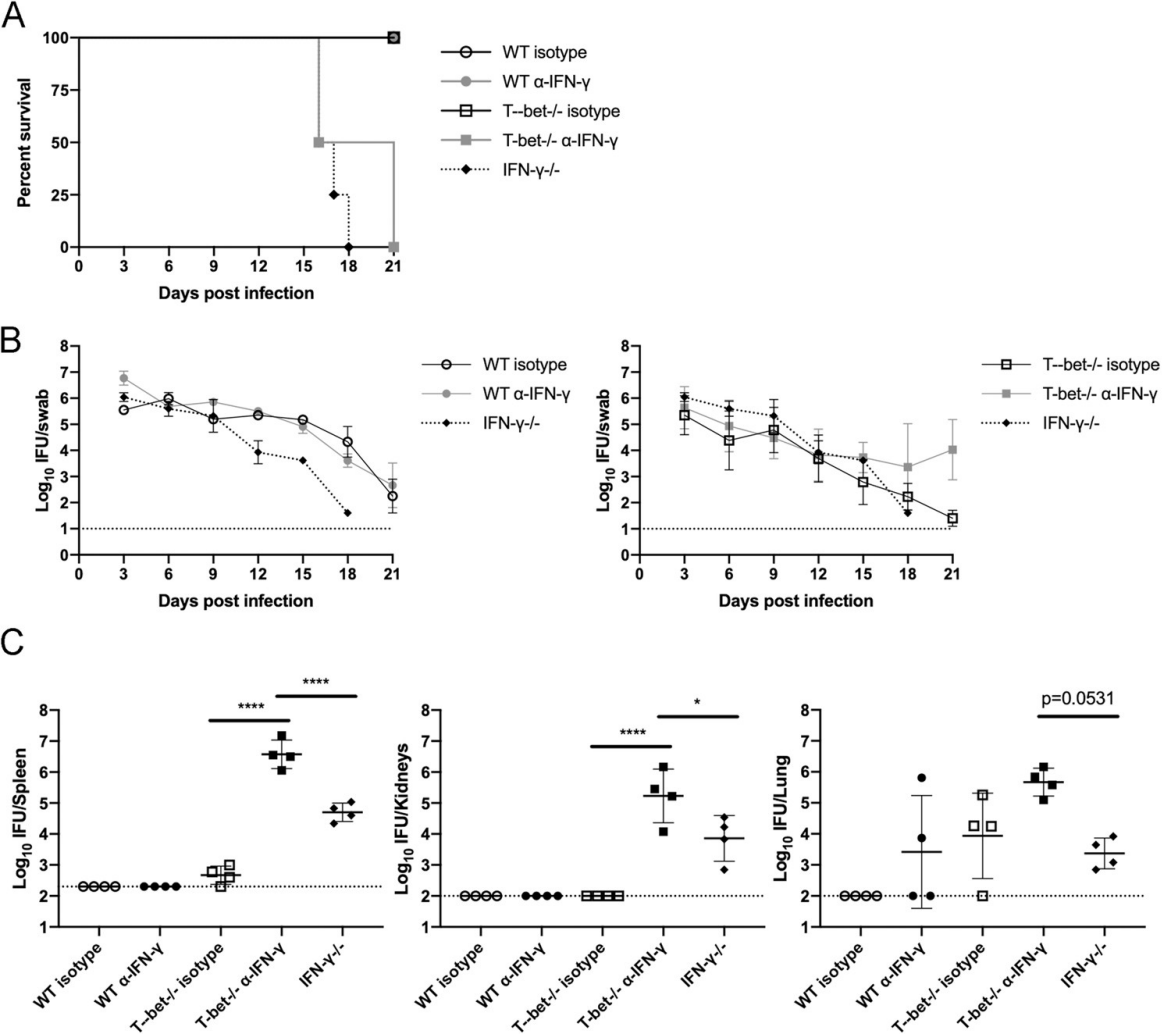
T-bet deficient mice given depleting α-IFN-ɣ are not able to control disseminated bacteria. Mice received 2.5mg Depo-Provera 7 days before infection with 1x10^5^ IFU *Chlamydia muridarum* i.vag. (A) Survival curve. (B) IFU per vaginal swab over time of mice from (A) Data has been split into two graphs for ease of visualization. Results are shown as mean with SEM. (C) Bacterial load per organ at time of euthanasia (1-way ANOVA). All mice surviving in WT α-IFN-ɣ, WT isotype, and T-bet-/- groups were euthanized as controls despite showing no signs of disease at day 21 when the last mice in the T-bet-/- α-IFN-ɣ were euthanized. n = 4 for all groups (A-C). Data is from one experiment.

Since T-bet-deficient mice lacking Th1 cells fully resolved *Chlamydia* infection of the FRT, it was of interest to determine whether this clearance required IFN-γ, since T-bet-independent pathways of CD4 IFN-γ production have been detected in other infection models [39]. Wild-type and T-bet-deficient mice were infected with *C*. *muridarum* and administered neutralizing antibody against IFN-γ or isotype control antibody. T-bet-deficient mice given anti-IFN-γ displayed signs of systemic disease around the same time as IFN-γ-deficient mice, approximately two weeks into infection. These symptoms included marked weight loss and decreased body conditioning and progressed until all mice in both of these groups had to be euthanized in compliance with IACUC regulations (Fig 6A). In contrast, mice in isotype control groups and wild-type administered anti-IFN-γ displayed no overt signs of systemic disease and survived until the end of the study. Interestingly, all groups exhibited similar shedding from the FRT (Fig 6B), consistent with earlier observations (Fig 5), suggesting again that IFN-γ signaling is not crucial to reducing bacterial burdens during initial FRT infection. To examine the extent of systemic dissemination of *Chlamydia* from the FRT, spleen, lung, and kidneys were examined for bacterial burdens either at the time of euthanasia or at 21 days for groups not showing symptoms of disease. As in IFN-γ-deficient mice, IFN-γ depletion by antibody treatment in T-bet-deficient mice led to increased dissemination (Fig 6C). In contrast, wild-type mice administered depleting antibody did not show the same degree of dissemination or systemic signs of disease (Fig 6C), suggesting their greater IFN-γ response was incompletely depleted by antibody. T-bet-deficient mice given isotype control antibody displayed detectable dissemination to the lung and spleen, but T-bet-deficient mice depleted for IFN-γ had significantly higher burdens than IFN-γ-deficient mice, suggesting T-bet further supports the immune response against disseminated bacteria outside of IFN-γ. Thus, in the absence of Th1 cells in T-bet-deficient mice, other sources of IFN-γ play a crucial role in protection from dissemination and death during *Chlamydia* infection.

## Discussion

Pathogen-specific CD4 Th1 cells develop from naïve precursors that are initially activated in the draining lymph node by dendritic cells bringing foreign antigen from the local site of infection [42]. These activating signals include ligation of the T cell receptor and CD28 by short peptides presented in the context host MHC class-II and CD80/CD86 on the surface of an appropriate dendritic cell [43]. Local cytokines (including IFN-γ and IL-12) allow these newly activated T cells to initiate programing events that commit to future secretion of IFN-γ upon secondary TCR ligation or non-cognate signals such IL-18 and IL-12 or TL1A [44,45]. These Th1 cells are able to combat a variety of intramacrophage infections via the production of local IFN-γ which causes macrophage activation and killing of internalized pathogens [32,41,46]. Key to the Th1 lineage commitment is the intracellular expression of the master Th1 transcription factor T-bet [25]. Thus, mice lacking T-bet are unable to resolve intramacrophage infections such as *Leishmania* or *Salmonella* [30,41,47].

Although *Chlamydia* is often referred to as a “Th1 pathogen” [10,11,21–23] due to prominent CD4 T cell IFN-γ production and the high susceptibility of IFN-γ-deficient mice, our data show that mice lacking T-bet can control FRT infection with essentially normal kinetics. Clearly, *Chlamydia muridarum* infection is not being controlled by classical T-bet Th1 cells in this mouse model. The involvement of *Chlamydia*-specific Th1 cells is further questioned by the absence of T-bet expression in CD4 T cells within the FRT during the period of infection control. Surprisingly, around 25% of CD4 T cells in the FRT of *Chlamydia*-infected T-bet-deficient mice also retained the capacity to produce IFN-γ. These data are reminiscent of the *M*. *tuberculosis* models where T-bet was not essential for protection, but CD4 T cells displaying a phenotype approximating a Th1 signature could mediate bacterial clearance [48]. Similarly, CD4 T cells in T-bet-deficient mice infected with *Toxoplasma gondii* retain the capacity to generate a prominent population of CD4 T cells producing IFN-γ, although these mice were still highly susceptible to infection [32]. Greater investigation of non-Th1 CD4 T cells in the *Chlamydia* model will be required in order to determine whether this population contributes significantly to pathogen clearance from the FRT.

Despite the high susceptibility of IFN-γ- and IFN-γR-deficient mice, it could be argued that the Th1 signature cytokine IFN-γ does not appear to play a prominent role in bacterial clearance within the FRT itself, since IFN-γ-deficient mice exert multiple logs of control on FRT shedding. The hypothesis that IFN-γ is largely irrelevant to FRT clearance in this model would also be supported by evidence that *Chlamydia* can escape IFN-γ-mediated mechanisms of defense. Previous work has shown that inclusions of the human pathogen *Chlamydia trachomatis* grown in mouse cells are susceptible to tagging by IFN-γ-induced immunity related GTPases that lead to destruction of inclusions, while *Chlamydia muridarum* escape these functions [49,50]. In addition, *Chlamydia trachomatis* contains a tryptophan synthase that allows escape of IFN-γ-induced reduction of available tryptophan via IDO expression in human host cells. *Chlamydia muridarum* is a tryptophan auxotroph that cannot escape the same IDO induction, though this is not induced in the mouse [51]. Together, these lines of evidence indicate that the respective *Chlamydial* species have adaptations specific to their natural host to escape IFN-γ-mediated defenses. *Chlamydia muridarum*, originally isolated out of a mouse host [52], retains host-specific adaptations to evade IFN-γ-mediated mechanisms in the murine host, while *Chlamydia trachomatis* as a human adapted pathogen may be able to mediate the similar evasion in the human host, but not in mice. In summary, there is insufficient evidence to show that IFN-γ producing CD4 T cells significantly contribute to the control of infection within the FRT during *C*. *muridarum* infection. However, it is important to note that there could still be a tissue protective role for IFN-γ that was unable to be assessed in our study due to high fatality rates [37]. Even if this was the case, any tissue protective role does not seem to require Th1 cells since T-bet-deficient mice do not display enhanced pathology.

In contrast, at sites outside of the FRT, IFN-γ consistently plays an essential role in limiting bacterial dissemination and preventing mortality. Mice lacking either IFN-γ or IFN-γR experience terminal disease with overt symptoms appearing within 2 weeks of vaginal infection. Similarly, neutralization of IFN-γ in T-bet-deficient mice caused a similar systemic infection, but did not affect wild-type mice presumably because their higher levels of IFN-γ were not fully depleted by antibody. The 100% mortality we observed in IFN-γ-deficient mice was greater than previous reports where evidence of persistent infection in surviving mice was detected. However, this disparity is likely explained by the use of different *Chlamydia* strains between these studies. Our laboratory uses the ATCC Weiss derivative which has been reported to have increased virulence over the Nigg strain used in prior studies [53]. Indeed, a recent paper by *Li et al*. described similar levels of fatality to our study and confirmed the primary importance of IFN-γ for systemic control of infection [54]. Interestingly, this same report also found that ILCs and NK cells were a major source of IFN-γ and prevented early systemic bacterial dissemination, a finding consistent with our observations of systemic spread in T-bet-deficient mice depleted of IFN-γ. Although the systemic anatomical niche of *Chlamydia* replication in the absence of IFN-γ is yet to be fully defined, it seems possible that tissue macrophages allow limited bacterial replication in the absence of innate IFN-γ. Macrophage M1 polarization, typically induced via IFN-γ signaling, has been shown to restrict intracellular *Chlamydia* replication while unpolarized or M2 macrophages allow growth. A role for innate IFN-γ working in concert with systemic antibody and possibly other phagocytes to prevent bacterial growth and dissemination has been suggested [33,55]. However, our data would argue that these mechanisms could be completely distinct from the mechanisms responsible for primary clearance within the FRT.

The question of what mechanism of protection exerts control of primary *Chlamydia* infection within the FRT remains unresolved. While IFN-γ-producing non-Th1 cells are present in the infected FRT, the evidence for local IFN-mediated protection is not entirely convincing. Similarly, we did not detect development of a Type-II response with IL-4 secreting CD4 T cells. Since *Chlamydia* replication is dependent on residence within a host cell inclusion, adaptive immune mechanisms involving cytotoxicity would be an attractive theoretical solution for control of intracellular replication. If *Chlamydia*-specific cytotoxic T cells could identify infected host epithelial cells and eliminate them before the completion of a 48-hour *Chlamydial* developmental cycle, progression of infection could be effectively curtailed. However, despite the conceptual appeal of this model, protection against *C*. *muridarum* within the FRT is CD4-dependent and mice lacking cytotoxic CD8 T cells control infection [13]. It is therefore tempting to speculate that *Chlamydia*-specific CD4 T cells gain the capacity for cytotoxic killing of the infected epithelium, a possibility previously raised by other laboratories examining Nitric oxide-dependent and Nitric oxide-independent mechanisms of epithelial cell killing [56–58]. An alternative or complementary mechanism might involve the recruitment of neutrophils via the development of Th17 cells. Indeed, we detected a sizable shift in T-bet-deficient mice towards CD4 T cells expressing RORγt and IL-17 expression. Enhanced Th17 responses have previously been associated with increased FRT pathology [37], but this was not evident in our study. These data might suggest that Th17 cells have been somewhat overlooked as a protective population in restraining local *Chlamydia* infection. Th17 type responses are typified by an influx of neutrophils that might phagocytose extracellular *Chlamydia* as well as support the barrier integrity of the mucosal epithelium. However, it should be noted that very low Th17 responses were detected in the FRT of wild-type mice perhaps suggesting that this subset is unlikely to be the major component of reproductive tract defense in the presence of T-bet. Studies are underway to examine the possibility of compensatory control of FRT infection by Th17 cells in the absence of T-bet. It is clear that further studies are required to pinpoint the precise mechanisms of CD4-mediated control of *Chlamydia* replication within the FRT.

In summary, our data show that, contrary to previous assumptions, classical T-bet-expressing Th1 cells are not required for clearance of primary infection with *C*. *muridarum* in the mouse FRT. In contrast, IFN-γ is essential to prevent fatal systemic proliferation of *Chlamydia*. In the absence of Th1 development within the FRT, we detected the emergence of T-bet-independent IFN-γ secreting CD4 T cells and a large population of Th17 cells, coincident with bacterial clearance without worsening pathology. Defining the contribution of CD4 T cell populations and others to immune-mediated clearance of *Chlamydia* will help further understanding of T cell-mediated bacterial clearance and FRT pathology.

## Methods

### Ethics statement

This study was carried out in strict accordance with the recommendations in the Guide for the Care and Use of Laboratory Animals of the National Institutes of Health. The University of California Davis is accredited by the Association for Assessment and Accreditation of Laboratory Animal Care (AAALAC). All animal experiments were approved by University of California Davis Institutional Animal Care and Use Committee (IACUC) (Protocol number 21869 and 22267).

### Mice

C57BL/6 (JAX stock no. 000664), MHC class II-deficient (JAX stock no. 003584), IFN-γ-deficient (JAX stock no. 002287), IFN-γ R1-deficient (JAX stock no. 003288), *Tbx21*-deficient (JAX stock no. 004648), CD4-Cre (JAX stock no. 017336), *Tbx21*f/f (JAX stock no. 022741), CD45.1 (JAX stock no. 002014), mice were purchased at 6–8 weeks old from The Jackson Laboratory (Bar Harbor, ME) and used for experiments at 7–12 weeks old. Breeding colonies for many of these strains were established at UC Davis and used in several experiments. All experiments were performed in agreement with regulations set by the University of California, Davis Institutional Animal Care and Use Committee.

### *Chlamydia* infection and burden

*Chlamydia muridarum* was purchased from ATCC and propagated as described previously [18]. The estrous cycle of the mice was synchronized via administration of 2.5mg of medroxyprogesterone acetate (Depo-Provera) subcutaneously one week prior to infection. For vaginal infections, 1x10^5^ IFU of *Chlamydia muridarum* was pipetted in SPG buffer into the vaginal vault of the mice. To assess in vivo bacterial shedding, vaginal swabs were taken and deposited into 500uL SPG buffer with two glass beads in 2mL microcentrifuge tubes. The tubes were shaken at 1400rpm for 5min at 4°C, after which the swab was removed. A dilution series of the bacterial suspension was used to infect a monolayer of HeLa cells in 96-well plates, allowed to grow into inclusions overnight, then fixed and stained for counting to determine IFU per swab. To determine IFU burden per organ, the organs of *Chlamydia muridarum* infected mice were homogenized in 1-2mL SPG. 1mL of the homogenate was shaken with glass beads at 1400rpm and 4°C for 5min, then centrifuged at 500g and 4°C and for 10min. The supernatant was isolated for use in the counting assay described above.

### Cytokine ELISAs

For ELISA quantification of IFN-γ production, CD4 T cells were isolated from the FRT of infected mice using a negative MACS CD4 T cell isolation kit on LS columns (Miltenyi Biotech). 2x10^4^ T cells were incubated overnight with 0.8x10^6^ irradiated splenocytes and 1x10^5^ heat-killed IFU *Chlamydia muridarum* elementary bodies per well, or α-CD3 (0.5μg/mL) and α-CD28 (1μg/mL) for controls. Culture supernatant was collected and ELISAs performed using the Invitrogen ELISA kit for IFN-ɣ, according to manufacturer’s instructions.

### *Salmonella* infection

*Salmonella enterica* Typhimurium strain BMM50 (SL1344 Δ*aroA*) was given to mice intravenously at a dose of 5x10^5^ CFU. A stock culture was streaked onto MacConkey agar and one colony was used to inoculate a culture grown overnight at 37°C in Luria-Bertani broth before dilution in PBS prior to administration.

### Flow cytometry

Lymphocytes were isolated from the female reproductive tract of mice 16 days post infection. The FRT was harvested into complete RPMI, diced, then incubated while stirring with collagenase IV (386mg/L MP Biomedicals) for 1 hour at 37°C. Cells were filtered (70μm cell strainer, Corning), then lymphocytes were isolated out of the supernatant using a Percoll gradient (GE Healthcare). Cells were stimulated with PMA (0.2 mM, Millipore Sigma) and Ionomycin (1ug/mL, Millipore Sigma) with Brefeldin A (71.4uM Millipore) for 3.5 hours at 37°C 5% CO_2_. Cells were stained for viability with Zombie Yellow (BioLegend) and surface markers B220-APC-eF780 (RA3-6B2, eBioscience), CD11b-APC-eF780 (M1/70, eBioscience), CD11c-APC-eF780 (N418, eBioscience), F4/80-APC-eF780 (BMB, eBioscience), CD4-PE (RM4-4, eBioscience), CD4-eF450 (RM4-5, eBioscience), CD8-PerCPCy5.5 (2.43, Tonbo), CD44-APC (IM7, eBioscience) CD62L-PETexasRed (MEL-14, Invitrogen), followed by intracellular stains IFN-γ-BV785 (XMG1.2, BioLegend), IL-4-PE (11B11, eBioscience), T-bet-PECy7 (4B10, eBioscience), RORγt-BV421 (Q31-378, BD Biosciences), and IL-17A-FITC (17B7, eBioscience) using the Foxp3 Transcription Factor Staining Kit (eBioscience). Data was acquired on an LSRFortessa (BD) and analyzed using FlowJo (Tree Star, San Carlos, CA). Contour plots are shown with 5% outliers.

### RNA-seq analysis

For each group, five samples were prepared by pooling three individual mice each. Lymphocytes were isolated from the FRT, as described above. CD4 T cells were enriched using a negative MACS CD4 T cell isolation kit on LS columns (Miltenyi Biotech). These cells were stained for subsequent FACS sorting using the viability stain Zombie Yellow (BioLegend) and antibodies for surface markers APC-B220 (RA3-6B2, eBioscience), APC-F4/80 (BM8.1, Tonbo Biosciences), APC-CD11b (M1/70, Tonbo Biosciences), APC-CD11c (N418, Tonbo Biosciences, eF450-CD4 (RM4-5, eBioscience), and PerCP-Cy5.5-CD8a (53–6.7, eBioscience). Events passing through the sequential gates for single cell, live, dump negative, and CD4+CD8- were collected and processed to isolate RNA (Qiagen RNeasy Mini Kit). Gene expression profiling was carried out using a 3’Tag-RNA-Seq protocol. Barcoded sequencing libraries were prepared using the QuantSeq FWD kit (Lexogen, Vienna, Austria) for multiplexed sequencing according to the recommendations of the manufacturer using also the UMI Second-Strand Synthesis Module (Lexogen). The fragment size distribution of the libraries was verified via micro-capillary gel electrophoresis on a Bioanalyzer 2100 (Agilent, Santa Clara, CA). The libraries were quantified by fluorometry on a Qubit fluorometer (LifeTechnologies, Carlsbad, CA), and pooled in equimolar ratios. Up to forty-eight libraries were sequenced per lane on a HiSeq 4000 sequencer (Illumina, San Diego, CA) with single-end 100 bp reads to 4–7 million reads per sample. Analysis of the sequencing data and differential gene expression was performed by UC Davis Bioinformatics Core. Raw reads were processed with HTStream v.1.1.0 (https://s4hts.github.io/HTStream/) to perform raw sequence data QA/QC, remove adapter contamination and low-quality bases/sequences. The trimmed reads were aligned to the *Mus musculus* GRCm38 primary assembly genome with GENCODE v.M23 annotation, using the aligner STAR v. 2.7.0f (Dobin, et al. 2013, Reference at https://www.ncbi.nlm.nih.gov/pubmed/23104886) to generate raw counts per gene. Differential expression analyses were conducted using limma-voom [59] (edgeR version 3.20.9, limma version 3.34.9, R version 3.4.4). The model used in limma included effects for treatment, RNA extraction batch, number of cells, and age at death. *Mus musculus* Ensembl gene identifiers and annotations were used in this study [60]. Raw data is available in NCBI’s Gene Expression Omnibus [61], and are accessible through GEO Series accession number GSE193909. S1 Table contains the full list of differentially expressed genes.

### Histopathology

Mice were euthanized at day 60 or 61 post infection and the FRT (vagina, uterus, oviducts, and ovaries) collected and immersion fixed in 10% neutral buffered formalin. The tissues were then embedded in paraffin and sectioned at 5μm before staining with hematoxylin and eosin. A board certified veterinary anatomic pathologist then analyzed and scored sections of each of the vagina, uterus, oviducts, and ovaries for acute inflammation (neutrophilic infiltration and edema), chronic inflammation (lymphohistiocytic infiltration), erosion (loss of mucosal epithelial cells regardless of breach of basement membrane), dilation (luminal distention), and fibrosis (increased fibroblasts or increased collagenous connective tissue). Ordinal scores on a 0 to 4 point scale reflect the severity and distribution of lesions. The cumulative lesion score for each individual is the sum of the scores for the oviduct and uterus.

### IFN-γ depletion *in vivo*

Mice were administered 0.2mg anti-IFN-γ antibody (R4-6A2, BioXCell) or isotype control antibody (Rat IgG1 anti-horseradish peroxidase, BioXCell) i.p. on days -1, 1, and every 3 days after infection with Chlamydia and monitored for bacterial shedding and the presence of bacteria in tissues at later time points.

### Statistics

Statistics were performed using GraphPad Prism version 8 (GraphPad Software, LLC). Either Mann-Whitney U test or one-way ANOVA were used. * p<0.05, ** p<0.01, *** p<0.01, and **** p<0.0001.

## Supporting information

S1 Fig T-bet deficient mouse models exhibit deficiencies during Salmonella infection and decreased Th1 staining. Mice received 5x105 CFU Salmonella i.v. (A) Bacterial burdens isolated from the spleen 11 days post infection. n = 3 for each group. Graph displays mean ± SD. (B) Example flow plots of T-bet and IFN-γ expression 11 days post infection. Cells are gated on lymphocytes, singlets, live cells, dump negative, and CD44hi CD62Llo. (C) Summary of plots from (B). n = 3 for all groups. Graphs depicts mea ± SD, 1-way ANOVA.
(DOCX)

S2 Fig CD8+ T cells from the FRT also exhibit a reduced capacity for IFN-γ production in T-bet deficient mice. Mice either received 2.5mg Depo-Provera 7 days before infection with 1x10^5^ IFU *Chlamydia muridarum* i.vag Lymphocytes were isolated from the FRT on day 17 post infection for *Chlamydia*-infected mice and stimulated with PMA/ionomycin in the presence of Brefeldin A before staining for flow cytometry. n = 4 for both groups. Cells are gated on the lymphocyte population, singlets, live cells, dump negative, and CD8+ CD4-. (A) Example flow plots of IFN-γ and CD44 expression. (B) Summary of (A). Graphs display mean ± SD, 1-way ANOVA.
(DOCX)

S1 Table Complete list of differential gene expression between Tbx21-/- and Wild-Type samples. Raw data is available in NCBI’s Gene Expression Omnibus and are accessible through GEO Series accession number GSE193909. LogFC is the log2 fold change with the Tbx21-/- samples as the numerator of the fold change. AveExpr is the average expression across all samples in log2 counts per million reads. P.Value is the raw p-value from the test that the log fold change differs from 0. Adj.P.Val is the Benjamini-Hochberg false discovery rate adjusted p-value.
(XLSX)
