# Supplementary material for: Th1 cells are dispensable for primary clearance of Chlamydia from the female reproductive tract of mice
Source: PLoS Pathog. 2022 Feb 23;18(2):e1010333. doi: 10.1371/journal.ppat.1010333 (PMC8901068; doi:10.1371/journal.ppat.1010333)
Supplement: S1 Fig — Mice received 5x10^5^ CFU Salmonella i.v. (A) Bacterial burdens isolated from the spleen 11 days post infection. n = 3 for each group. Graph displays mean ± SD. (B) Example flow plots of T-bet and IFN-γ expression 11 days post infection. Cells are gated on lymphocytes, singlets, live cells, dump negative, and CD44hi CD62Llo. (C) Summary of plots from (B). n = 3 for all groups. Graphs depicts mea ± SD, 1-way ANOVA. (DOCX) [file ppat.1010333.s001.docx]

**S1 Fig: T-bet deficient mouse models exhibit deficiencies during *Salmonella* infection and decreased Th1 staining.** Mice received 5x10^5^ CFU *Salmonella* i.v. (A) Bacterial burdens isolated from the spleen 11 days post infection. n=3 for each group. Graph displays mean ± SD. (B) Example flow plots of T-bet and IFN-γ expression 11 days post infection. Cells are gated on lymphocytes, singlets, live cells, dump negative, and CD44hi CD62Llo. (C) Summary of plots from (B). n=3 for all groups. Graphs depicts mea ± SD, 1-way ANOVA.
