# Supplementary material for: Th1 cells are dispensable for primary clearance of Chlamydia from the female reproductive tract of mice
Source: PLoS Pathog. 2022 Feb 23;18(2):e1010333. doi: 10.1371/journal.ppat.1010333 (PMC8901068; doi:10.1371/journal.ppat.1010333)
Supplement: S2 Fig — Mice either received 2.5mg Depo-Provera 7 days before infection with 1x10^5^ IFU Chlamydia muridarum i.vag Lymphocytes were isolated from the FRT on day 17 post infection for Chlamydia-infected mice and stimulated with PMA/ionomycin in the presence of Brefeldin A before staining for flow cytometry. n = 4 for both groups. Cells are gated on the lymphocyte population, singlets, live cells, dump negative, and CD8+ CD4-. (A) Example flow plots of IFN-γ and CD44 expression. (B) Summary of (A). Graphs display mean ± SD, 1-way ANOVA. (DOCX) [file ppat.1010333.s002.docx]

**S2 Fig: CD8+ T cells from the FRT also exhibit a reduced capacity for IFN-γ production in T-bet deficient mice.** Mice either received 2.5mg Depo-Provera 7 days before infection with 1x10^5^ IFU *Chlamydia muridarum* i.vag Lymphocytes were isolated from the FRT on day 17 post infection for *Chlamydia*-infected mice and stimulated with PMA/ionomycin in the presence of Brefeldin A before staining for flow cytometry. n=4 for both groups. Cells are gated on the lymphocyte population, singlets, live cells, dump negative, and CD8+ CD4-. (A) Example flow plots of IFN-γ and CD44 expression. (B) Summary of (A). Graphs display mean ± SD, 1-way ANOVA.
